# Correlations between the Total Antioxidant Activity and Biochemical Parameters of Cow Milk Depending on the Number of Somatic Cells

**DOI:** 10.1155/2022/5323621

**Published:** 2022-01-13

**Authors:** Sergei Yu. Zaitsev, Oksana A. Voronina, Anastasia A. Savina, Larisa P. Ignatieva, Nadezhda V. Bogolyubova

**Affiliations:** Federal Research Center for Animal Husbandry named after Academy Member L.K. Ernst, Dubrovitsy 60, Podolsk Municipal District, Moscow Region 142132, Russia

## Abstract

The aim of the work was to study the correlations between the total amount of water-soluble antioxidants (TAWSA) and biochemical parameters (BC) of cow milk depending on the somatic cell count (SCC). The BC and TAWSA values of cow milk were measured by spectroscopic and amperometric methods, respectively. The milk samples from the black-and-white cows (Moscow region) were divided according to SCС values: (1) ≤200, (2) 200-499, (3) 500-999, and (4) ≥1000 thousand units/mL. The average TAWSA values for groups 1, 2, 3, and 4 (33, 15, 13, and 12 milk samples) were the following: 15.95 ± 0.74, 14.45 ± 0.84, 16.04 ± 0.63, and 14.58 ± 1.18. The correlations between TAWSA and BC (group 1) were the following: total fat percentage (TFP) -0.305; true protein percentage (TP1) -0.197; total nitrogen percentage (TN2) -0.210; lactose -0.156; solids-not-fat (SNF) -0.276; total dry matter (TDM) -0.399; freezing point (FP) -0.112; pH -0.114; somatic cell count (SCC) - (-0,052). The correlations between TAWSA and BC (group 2) were the following: TFP -0.332; TP1 -0.296; TN2 -0.303; lactose - (-0.308); SNF -0.159; TDM -0.391; FP -0.226; pH - (-0.211); SCC -0.193. The correlations between TAWSA and BC (group 3) were the following: TFP - (-0.352); TP1 - (-0.411); TN2 – (-0.401); lactose - (-0.166); SNF - (-0.462); TDM - (-0.504); FP - (-0.766); pH - (-0.047); SCC - (-0.698). The correlations between TAWSA and BC (group 4) were the following: TFP -0.159; TP1 -0.046; TN2 – 0.077; lactose - (-0.317); SNF - (-0.237); TDM -0.058; FP - (-0.036); pH - (-0.477); SCC - (-0.072). These data are important in assessing the physiological-biochemical status and state of the antioxidant defense system of cows' organism.

## 1. Introduction

Milk is an important biological fluid and intensively studied in many aspects: from fundamental interfacial problems (of such complex colloid system) to the properties and quality of milk production. A comprehensive analysis of the biochemical properties of animal milk should include the study of the antioxidant activity (AOA) of such animal fluids, which is associated with the formation of reactive oxygen species (ROS). It is well known that ROS are formed during many metabolic processes in biological fluids, having a negative effect on the main biologically active compounds (BAC), on organs and tissues, etc. [1–3]. There are many reasons for the high interest in the study of the total amount of water-soluble antioxidants (TAWSA) as indicators of the antioxidant defense system against ROS. For example, the AOA data are a valuable source of information “on the state of health and the level of stress resistance of humans and productive animals in a farm environment” [4]. This is due to the search for “functional products” in the system of “antioxidant nutrition” in humans and in animal feeding [4]. Such attention to the study of the antioxidant defense system has led to development a few methods for studying its work [5–7].

Here, we will focus only on the electrochemical method, which is associated with the amperometric detection of the oxidation reaction signal [5–7], because we are not being able to list all known methods for studying AOA and biochemical parameters of cow milk. Amperometric detection is widely used as one of the reliable, available, and easy-to-use methods for the determination of antioxidants [5–7]. It is well known that the magnitude of the electric current depends on the nature of the “analyte,” the material of the working electrode, the potential applied to the electrode, etc. [5–7]. The sensitivity of the amperometric detector is very high due to low noise levels, of the order of 10-12 A [5–7]. Methods and approaches to the determination and analysis of the main biochemical parameters of cows' milk are described in a large number of works (only a few of which will be cited here [8–11]). In addition, the main of these modern methods is included in the corresponding Russian state standards (GOST) and technical requirements (TR) [12–14].

According to the requirements of the technical regulations for milk and dairy products of the Eurasian Economic Union [12], which came into force in January 2020, it is necessary to monitor the somatic cell count (SCC), i.e., the level of somatic cells, in raw milk [12]. For a healthy animal, the number of cells does not exceed 200 thousand cells in 1 mL of milk sample (2 · 10^5^ cells/cm^3^) from one cow [12]. In contrast, the SCC values in the “raw milk” (so-called “raw material” [13]) must be below 4 · 10^5^ cells/cm^3^ according to the Russia state standards [13, 14], which are still valid.

Somatic milk cells [15, 16] are roughly represented by epithelial cells of the mammary glands, alveoli, and small milk ducts; degenerated epithelial cells with a dilapidated structure; and blood cells (lymphocytes, neutrophils, eosinophils, etc.) [17]. Flow cytometry analysis allows differentiating of somatic cell populations in milk [15]. From the practical point of view, the typing of milk leukocytes seems to be the most valuable at farms [16]. This is an important step in understanding the immune response of the udder [16], the immunological biology of milk and mammary glands [17], etc. The number of somatic cells is kept as constant as possible in healthy animals [16, 17], whereas the inflammatory process in the udder is accompanied by an increase in the total number of neutrophils and lymphocytes [16].

The somatic cell count (SCC), i.e., the total number of somatic cells in milk, depends on many factors: breed of animals, age, lactation stage, animal health status, and number of calves. As soon as the process of inflammation appears in the udder, SCC values increase sharply by an order of magnitude, and sometimes, even higher. Primary inflammatory processes, even without visible symptoms, can be detected by an increase in the total number of somatic cells. There are only minor changes in milk biochemical parameters observed at so-called “normal” level of somatic cells, whereas the increased SCC values (released due to the inflammatory process) bring the milk uncharacteristic taste and odor. The milk obtained in the last case is not suitable for further processing and often causes distortions in the technological chain of dairy product preparations. In the most serious situations, such milk can directly contain pathogens (staphylococci, etc.), which can infect consumers with some diseases. For example, “mastitis” milk causes food poisoning of bacterial origin, disorders of the gastrointestinal tract, and streptococcal sore throat in adults and, especially, for children.

In this regard, it is of great importance to study the correlations of the main biochemical parameters of cow's milk samples with certain values of the total amount of water-soluble antioxidants (TAWSA). For example, in the work of Arab scientists in 2008 [18], the indicators of the total number of somatic cells and their three types (macrophages, lymphocytes, and polymorphonuclear leukocytes), as well as the antioxidant activity of the following enzymes, catalase, superoxide dismutase, and glutathione peroxidase, were studied in detail. The authors [18] randomly selected 43 cow milk samples (from 8 farms in Tunisia from November 2005 to February 2006). All samples were separated by the authors [18] into three groups in accordance with the number of SCC: less than 1000 thousand units/mL, from 1000 to 1500 thousand units/mL; more than 1500 thousand units/mL in comparison to the average over the sum of all samples [18]. The authors [18] found that catalase and glutathione peroxidase correlated at a high degree (about 0.66) with both the total number of somatic cells and the number of neutrophils. From a biochemical point of view, the product of the catalase reaction is hydrogen peroxide and a hydroxyl radical [9]; the activity of these enzymes increases precisely to neutralize the negative effect of ROS [18]. In addition, the total activity of these enzymes is one of the most important markers of mastitis in cows [18], i.e., a promising indicator of milk quality and health of cows [18–20].

The major points mentioned above, especially the relationships between the total antioxidant activity and biochemical parameters of cow milk at various somatic cell counts, have been little investigated in all of their complexity.

The aims of the work were the following: to measure the total amount of water-soluble antioxidants and some other biochemical parameters of cow milk depending on the somatic cell count in the whole range of values; to analyze the statistics; and to establish the correlations between the obtained parameters of cow milk.

## 2. Materials and Methods

Milk samples (total amount of 73 units) of black-and-white cows were obtained from farms of the Moscow region during the winter stall period. The experimental protocols (concerning these animals) were approved by the Bioethical Committee of the Federal Research Center for Animal Husbandry named after Academy Member L.K. Ernst. All experiments and conditions (animal care, feeding, biological material sampling, etc.) were fulfilled in accordance with the applicable regulations (internationally recognized guidelines and local acts).

Methods and approaches to the determination and analysis of the main biochemical parameters of cow milk (fat, proteins, lactose, etc.) were described in a large number of works [8–14]. In our research, the analytical system MilkoScan 7/Fossomatic 7 DC (Denmark) was used to analyze the component composition of cow's milk and the somatic cell count (SCC). MilkoScan 7 is a spectrophotometer based on Fourier transform infrared spectrophotometry. Fossomatic 7 performs somatic cell counting based on flow cytometry.

The amperometric method [5–7] was used to study the total amount of water-soluble antioxidants (TAWSA). The measurements were carried out on a TsvetYauza 01-AA device [5, 10–12]. The TAWSA was determined by measuring the strength of the electric current arising during the oxidation of molecules on the surface of the working electrode at a certain potential. The “working solutions” of gallic acid (100 mg/dm^3^) were used as a standard for measuring the TAWSA of the samples, which is described in detail in [5–7]. Phosphoric acid solution 0.0022 mol/dm^3^ was used as an eluent. The flow rate of the eluent to the peristaltic pump is 1.2 cm^3^/min. Since the signal from the analyzed milk samples exceeded the signal of the calibration solution (4.0 mg/dm^3^ gallic acid), the samples were preliminarily diluted (100 *μ*L of the sample plus 1900 *μ*L of bidistilled water). At the same time, the key to the reliability and good repeatability of the obtained result was the cleanliness of the working surface of the electrode and reliable calibration at the beginning of each working procedure. The calculation of the mass concentration of antioxidants (*X*, mg/g) was performed in equivalent to gallic acid, taking into account the dilution of the sample, according to the following equation [5–7].

(1) $$\begin{array}{c} X=\frac{\left(X_{G}\cdot N\cdot V_{n}\right)}{\left(m_{n}\cdot 1000\right)}, \end{array}$$

where *X*_*G*_ is the mass concentration of antioxidants (measured in mg/L);

*N* is the dilution factor of the analyzed sample;

*V*_*n*_ is the volume of the solution (extract) in the analyzed sample (in mL);

*m*_*n*_ is the sample of the “analyte” (in g).

The results of the general antioxidant activity and the main biochemical parameters of cow's milk were statistically processed using the “Microsoft Excel” program.

## 3. Results and Discussion

Important data on the biochemical composition and antioxidant activity of milk samples from black-and-white cows of the Moscow region in the “winter stall period” were obtained. At the first stage of the analysis, all indicators for 73 milk samples of black-and-white cows from the Moscow region farms were evaluated. The TAWSA values for all milk samples ranged from 6.8 mg/g to 27.9 mg/g (mean value 15.43 ± 3.7 mg/g). Relatively weak correlations were found between TAWSA and the following milk parameters for all these samples: total fat percentage (TFP) - 0.27; true protein percentage (TP1) and total nitrogen percentage (TN2) - – 0.18 (in both cases); lactose - 0.14; solids-not-fat (SNF) - 0.14; total dry matter (TDM) - 0.28; freezing temperature point (FP) - 0.03; pH - (-0.16); the somatic cell counts (SCC) - (-0.01).

At the second stage of the analysis, the TAWSA and the average biochemical parameters were assessed for four groups of cows, which were selected according to the values of the SCC. Such a division was fulfilled in accordance with the requirements of the technical regulations for milk and dairy products of the Eurasian Economic Union and the corresponding Russian state standards and technical requirements [12–14]. That is why four groups were selected according to SCC values: (1) less than 200 thousand units/mL, (2) from 200 to 499 thousand units/mL, (3) from 500 to 999 thousand units/mL, and (4) more than 1000 thousand units/mL for milk samples of black-and-white cows of the Moscow region farms (as raw materials).

Group 1 (selected according to SCC values less than 200 thousand units/mL) was the most numerous of the four and included 33 milk samples of black-and-white cows of the Moscow region farms (Table 1).

The TAWSA values of the 33 milk samples of group 1 ranged from 6.80 mg/g to 27.91 mg/g, and the average value was 15.95 ± 0.74 mg/g (Table 1). The correlations between TAWSA and the following parameters of milk were the following: total fat percentage (TFP) -0.305; true protein percentage (TP1) -0.197; total nitrogen percentage (TN2) -0.210; lactose -0.156; solids-not-fat (SNF) -0.276; total dry matter (TDM) -0.399; freezing point (FP) -0.112; pH -0.114; somatic cell count (SCC) - (-0,052). Average values were obtained for the following parameters: TFP - 5.16%; TP1 or TN2 - 3.22% or 3.45%; lactose - 4.87%; SNF - 9.16%; TDM - 14.37%; freezing point – (-0.540)°С; pH - 6.57; SCC – about 99 thousand units/mL.

Group 2 (selected according to SCC values from 200 to 499 thousand units/mL) consisted of 15 milk samples of black-and-white cows of the Moscow region farms (Table 2).

The TAWSA values of the 15 milk samples of group 2 ranged from 8.90 mg/g to 18.99 mg/g, and the average value was 14.45 ± 0.84 mg/g. The average values obtained for the following parameters: TFP - 4.72% (*р* < 0.1 to group 1); TP1 and TN2-3.26% and 3.49%; lactose - 4.80%; SNF - 9.14%; total dry matter - 13.92% (*р* < 0.05 to group 1); freezing point – (-0.539)°C; pH - 6.53 (*р* < 0.05 to group 1); SCC - about 333 thousand units/mL (*р* < 0.01 to group 1). The correlations between TAWSA and the following parameters of milk were the following: TFP -0.332; TP1-0.296; TN2 - 0.303; lactose - (-0.308); SNF -0.159; TDM -0.391; FP – (-0.226); pH - (-0.211); SCC -0.193.

Group 3 (selected according to SCC values from 500 to 999 thousand units/mL) consisted of 13 milk samples of black-and-white cows of the Moscow region farms (Table 3).

The TAWSA values of the 13 milk samples of the group 3 ranged from 13.60 mg/g to 22.25 mg/g, and the average value was 16.05 ± 0.63 mg/g. The average values obtained for the following parameters: TFP - 5.40%; TP1 and TN2-3.58% and 3.81% (for both, *р* < 0.05 to group 1); lactose - 4.51% (*р* < 0.01 to group 1); SNF - 9.21%; total dry matter - 14.67%; freezing point – (-0.540)°С; pH - 6.54 units; SCC – 805.23 thousand units/mL (*р* < 0.01 to group 1). The correlations between TAWSA and the following parameters of milk were found: MFF - (-0.353); TP1 and TN2 - (-0.411) and (-0.401); lactose - (-0.166); SNF - (-0.472); TDM - (-0.504); FP - 0.766; pH - (-0.047); SCC - (-0.725).

Group 4 (selected according to SCC values more than 1000 thousand units/mL) consisted of 12 milk samples of black-and-white cows of the Moscow region farms (Table 4).

The TAWSA values for these milk samples of group 4 ranged from 5.80 mg/g to 20.30 mg/g (mean value 14.58 ± 1.18 mg/g). The average values obtained for the following parameters: TFP - 5.18%; TP1 and TN2-3.38% and 3.61% (*р* < 0.1 to group 1); lactose - 4.47% (*р* < 0.01 to group 1); SNF - 8.93% (*р* < 0.1 to group 1); dry matter - 14.20%; freezing point - 0.534°С (*р* < 0.1 to group 1); pH - 6.52 (*р* < 0.1 to group 1); SCC - 3259 thousand units/mL (*р* < 0.01 to group 1). The correlations between TAWSA and BC (group 4) were the following: TFP -0.159; TP1-0.100; TN2 – 0.048; lactose - (-0.317); SNF - (-0.237); TDM -0.058; FP - (-0.036); pH - (-0.477); SCC - (-0.072).

Thus, there are almost maximal (7 from 9) significant correlations between TAWSA and the major milk parameters for group 3 (i.e., 3 strong, 4 moderate, 1 relatively weak, 1 weak). There are 5 moderate and 4 relatively weak correlations (from 9 total) between TAWSA and the major milk parameters for group 2. There are only 3 or 2 moderate, 5 or 2 relatively weak, and 1 or 5 weak correlations between TAWSA and the major milk parameters for groups 1 or 4, respectively. As shown in our previous publications on the study of correlations between TAWSA and biochemical parameters of animal blood [7, 21], positive or negative values of correlations are not as important as their absolute values. These correlations can be very strong (0.75-0.99), strong (0.50-0.74), moderate (0.25-0.49), or weak (0.01-0.24) according to their absolute values [21]. In the last case (weak correlations), it makes sense to discuss only correlations above 0.1 (i.e., from 0.10 to 0.25) that can be informally assigned as “relatively weak.” For example, moderate (-0.308) or relatively weak (-0.166) correlations between TAWSA and such biochemical parameter as lactose were found for groups 2 or 3, respectively. The most important correlations (in the case of cow milk) between TAWSA and such biochemical parameters as total fat, true protein and total nitrogen percentages were found for group 2 (or group 3), i.e., TFP -0.332 (or -0.352); TP1 and TN2 -0.296 (or -0.411) and 0.303 (or -0.401), respectively. It is important to highlight the strong correlations between TAWSA and such biochemical parameters as total dry matter; freezing point and somatic cell count were found only in the case of group 3, i.e., TDM - (-0.504); FP - (-0.766) and SCC - (-0.725), as well as moderate correlation between TAWSA and SNF - (-0.472).

It is well-known that an increased SCC values lead to some changes in the chemical composition of milk, for example, a decrease in titration acidity and an increase of the milk pH. These effects can lead to a change in the redox potential of milk [22]. However, in our study, the pH practically did not change and remained in the range from 6.57 (group 1) to 6.53 (groups 2 and 3) and 6.52 (group 4). From a practical point of view, it is important to check a relationship between a milk yield and the content of somatic cells in the case of each of the group, mentioned above. Usually in this case, the milk yield of cows is significantly reduced [23]. We found that for the cow groups 1-4, the milk yield values were the following: 22.9 ± 5.9 L, 20.7 ± 7.8 L, 17.9 ± 8.8 L, and 19.3 ± 8.9 L, respectively. When analyzing the milk yield by these groups, one can observe a gradual decrease in the amount of milk yield by increasing the number of somatic cells. In addition, the correlations between TAWSA and the milk yield values were the following: -0.325, -0.136, -0.107, and -0.144. Thus, only a moderate correlation between milk yield and SCC was found in the case of the group 1 (-0.325), while only weak negative correlations were found for groups 2-4.

It is important to highlight that in the literature, there is only a single indication of the correlation between total antioxidant activity (determined by another method) and the total milk yield throughout the large herd with a coefficient of -0.22 [24]. This value (-0.22) [24] corresponds, in general, to our abovementioned correlations by sign and number when averaged over all groups. Moreover, in the case of group 1, we found significantly higher correlations between the values of TAWSA and average milk yield, which confirmed the importance of limiting the SCC number below 200 thousand units/mL.

All correlations mentioned above (especially the strong and moderate), will give valuable information for future prognosis of milk technological properties and quality in addition to the standard values.

## 4. Conclusions

From the abovementioned results, it is possible to distinguish not only the contribution of each indicator to the milk raw materials but also the correlations between TAWSA and the component composition of cow's milk (revealed for the first time). It is important to highlight the strong and moderate correlations found between TAWSA and the majority of the biochemical parameters in the case of groups 1-3. The weakest correlation between TAWSA and most of the above parameters of cow milk found for group 4 could be associated with the maximum SCC values.

Taking into account that the absolute values of the majority of biochemical and physical-chemical parameters of milk do not change significantly (even nonlinearly) with an increase in the SCC values, the obtained correlation coefficients can be an important element of milk research. These data complement with the known models and can be useful for a more detailed analysis of animal milk, assessment of the animal physiological and biochemical status, and dairy product quality.

## Figures and Tables

**Table 1:** The major biochemical parameters and component composition of milk samples (*n* = 33, group 1).

Biochemical parameters	Average value	Mean	Min	Max	CV, %	SD	±m
TFP, %	5.16	4.93	3.62	7.30	15.88	0.82	0.14
TP1, %	3.22	3.13	2.65	4.49	12.74	0.41	0.07
TN2, %	3.45	3.38	2.89	4.75	11.96	0.41	0.07
TAWSA mg/g	15.95	16.00	6.80	27.91	26.72	4.26	0.74
Lactose, %	4.87	4.90	4.51	5.32	3.98	0.19	0.09
SNF, %	9.16	9.11	8.24	10.80	5.38	0.49	0.09
TDM, %	14.37	14.11	12.68	19.93	6.90	0.99	0.17
FP, °С	-0.540	-0.538	-0.568	-0.526	-1.77	0.01	0.01
pH, units	6.57	6.56	6.47	6.84	1.07	0.07	0.01
SCC, 10^3^ cells/mL	99.33	73	27	194	56.64	52.87	9.20

**Table 2:** The major biochemical parameters and component composition of milk samples (*n* = 15, group 2).

Biochemical parameters	Average value	Mean	Min	Max	CV, %	SD	±m
TFP, %	4.72	4.84	3.61	6.00	15.44	0.73	0.19
TP1, %	3.26	3.31	2.60	3.78	9.00	0.29	0.08
TN2, %	3.49	3.53	2.84	4.03	8.51	0.30	0.08
TAWSA mg/g	14.45	14.81	8.90	18.99	22.64	3.27	0.84
Lactose, %	4.80	4.79	4.28	5.23	4.51	0.22	0.06
SNF, %	9.14	9.14	8.36	9.63	3.55	0.32	0.02
TDM, %	13.92	14	12.28	14.8	5.28	0.73	0.19
FP, °С	-0.539	-0.541	-0.556	-0.519	-1.91	0.01	0.00
pH, units	6.53	6.52	6.42	6.67	0.98	0.06	0.02
SCC, 10^3^ cell/mL	333.33	299	208	499	28.61	95.36	24.62

**Table 3:** The major biochemical parameters and component composition of milk samples (*n* = 13, group 3).

Biochemical parameters	Average value	Mean	Min	Max	CV, %	SD	±m
TFP, %	5.40	5.35	4.06	6.84	15.26	0.82	0.23
TP1, %	3.58	3.49	2.48	4.57	17.32	0.62	0.17
TN2, %	3.81	3.72	2.71	4.81	16.66	0.64	0.18
TAWSA mg/g	16.05	15.40	13.60	22.25	14.12	2.27	0.63
Lactose, %	4.51	4.55	3.51	5.14	9.52	0.43	0.12
SNF, %	9.21	9.20	7.92	10.60	8.48	0.78	0.22
TDM, %	14.67	14.72	12.72	17.02	9.07	1.33	0.37
FP, °С	-0.540	-0.544	-0.55	-0.519	-0.78	0.01	0.01
pH, units	6.54	6.52	6.42	6.68	1.33	0.09	0.02
SCC, 10^3^ cell/mL	805.23	903	542	973	19.75	159.0	44.10

**Table 4:** The major biochemical parameters and component composition of milk samples (*n* = 12, group 4).

Biochemical parameters	Average value	Mean	Min	Max	CV, %	SD	±m
TFP, %	5.18	4.96	3.80	6.97	17.63	0.91	0.26
TP1, %	3.38	3.41	2.38	4.24	16.65	0.56	0.16
TN2, %	3.70	3.65	3.01	4.48	12.86	0.48	0.14
TAWSA mg/g	14.58	13.64	5.80	20.30	27.98	4.08	1.18
Lactose, %	4.47	4.55	3.63	5.04	9.32	0.42	0.12
SNF, %	8.93	8.88	8.44	9.79	4.82	0.43	0.12
TDM, %	14.20	14.05	12.71	16.27	6.11	0.87	0.25
FP, °С	-0.530	-0.537	-0.554	-0.517	-2.24	0.01	0.01
pH, units	6.52	6.53	6.36	6.65	1.30	0.09	0.02
SCC, 10^3^ cell/mL	3259.11	2614	1004	7493	64.89	2115	705

## Data Availability

The data supporting the reported results can be found at https://www.vij.ru/institut/struktura-organizatsii/nauchnye-podrazdeleniya/52-gruppa-analiticheskoj-biohimii.

## Supplementary Materials

Supplementary Materials Supplementary Materials will be mentioned as Tables S1-S4.
