# Supplementary material for: Correlations between the Total Antioxidant Activity and Biochemical Parameters of Cow Milk Depending on the Number of Somatic Cells
Source: Int J Food Sci. 2022 Jan 13;2022:5323621. doi: 10.1155/2022/5323621 (PMC8776491; doi:10.1155/2022/5323621)
Supplement: Supplementary Materials — will be mentioned as Tables S1-S4. [file 5323621.f1.pdf]

Supplementary Materials to the manuscript 5323621

Correlations between the total antioxidant activity and biochemical parameters of cow milk depending on the number of somatic cells

Sergei Yu. Zaitsev, Oksana A. Voronina, Anastasia A. Savina, Larisa P. Ignatieva, Nadezhda V. Bogolyubova

Table S1. The main biochemical parameters and component composition of milk samples from 33 black-and-white cows (group 1).

№	TFP, %	TP1, %	TN2, %	TAWSA mg/g	Lactose, %	SNF, %	TDM, %	FP, ºC	pH, units	SCC, 10^3 cell/ml
1	4.25	3.07	3.28	13.50	4.97	9.11	13.38	-0.534	6.68	27
2	6.21	2.88	3.12	6.80	4.79	8.64	14.96	-0.533	6.5	39
3	4.95	3.22	3.46	9.40	4.94	9.21	14.23	-0.552	6.61	64
4	4.32	3.12	3.35	13.30	5.04	9.18	13.57	-0.538	6.63	131
5	6.49	2.75	2.99	11.00	4.72	8.61	15.12	-0.544	6.54	52
6	4.78	3.52	3.75	16.00	4.85	9.46	14.33	-0.543	6.52	153
7	6.62	3.89	4.13	21.30	4.59	9.66	16.32	-0.546	6.47	98
8	4.64	3.03	3.24	15.20	5.32	9.39	13.98	-0.555	6.64	33
9	5.61	4.49	4.75	19.40	5.05	10.8	16.57	-0.562	6.84	120
10	4.83	3.23	3.45	13.30	4.6	8.82	13.71	-0.539	6.58	192
11	5.03	2.65	2.89	27.91	5.04	8.67	13.83	-0.527	6.61	37
12	7.01	3.21	3.49	22.07	4.61	8.99	16.1	-0.537	6.51	43
13	4.71	3.62	3.84	17.83	4.88	9.57	14.35	-0.529	6.53	194
14	4.98	3.46	3.68	16.24	4.95	9.57	14.57	-0.539	6.56	165
15	3.62	3.04	3.23	16.19	5.01	9.11	12.68	-0.536	6.58	44
16	4.77	2.83	3.08	16.45	4.93	8.77	13.62	-0.543	6.56	182
17	4.43	3.38	3.62	14.73	5	9.49	14	-0.536	6.55	82
18	5.31	3.06	3.3	18.24	4.96	9.12	14.52	-0.540	6.55	54
19	4.88	2.84	3.04	13.78	4.73	8.6	13.45	-0.536	6.61	46
20	4.93	3.13	3.38	17.31	4.82	9.02	14.11	-0.538	6.51	73
21	4.98	2.67	2.9	15.81	4.99	8.7	13.68	-0.543	6.61	49
22	5.64	3.06	3.31	20.92	5.04	9.21	14.82	-0.556	6.6	68

23	7.30	3.22	3.45	20.26	5.18	9.56	16.93	-0.568	6.58	119
24	5.77	3.53	3.76	21.03	5.07	9.76	15.52	-0.543	6.63	128
25	4.71	3.02	3.24	15.44	4.73	8.76	13.57	-0.530	6.57	70
26	4.90	3.94	4.19	18.97	4.84	9.95	14.93	-0.538	6.51	110
27	4.73	3.34	3.56	8.91	4.9	9.34	14.11	-0.541	6.53	182
28	4.74	3.4	3.61	13.38	4.64	9.1	13.79	-0.537	6.6	109
29	5.86	2.68	2.91	16.85	4.53	8.24	14.08	-0.529	6.49	42
30	4.45	3.12	3.34	10.77	4.89	9.04	13.5	-0.535	6.55	73
31	5.11	2.72	2.97	13.79	4.92	8.62	13.83	-0.537	6.5	27
32	4.50	3.26	3.5	13.99	4.61	8.97	13.52	-0.535	6.49	146
33	5.20	3.77	3.98	16.16	4.51	9.4	14.66	-0.526	6.52	128

Table S2. The main biochemical parameters and component composition of milk samples from 15 black-and-white cows (group 2).

**№**	**TFP, %**	**TP1, %**	**TN2, %**	**TAWSA mg/g**	**Lactose, %**	**SNF, %**	**TDM, %**	**FP, °C**	**pH, units**	**SCC, 10^3 cell/ml**
1	4.37	3.42	3.63	8.90	4.61	9.06	13.51	-0.519	6.57	243
2	5.28	3.18	3.41	11.90	4.87	9.14	14.56	-0.541	6.61	348
3	5.52	2.96	3.19	13.10	5.23	9.26	14.8	-0.556	6.67	291
4	5.36	3.78	4.03	18.99	4.28	9.15	14.61	-0.529	6.42	408
5	4.50	3.45	3.69	17.44	4.74	9.35	13.97	-0.538	6.52	244
6	4.84	3.31	3.53	15.83	4.67	9.1	13.98	-0.536	6.51	208
7	3.61	3.19	3.42	14.35	4.84	9.11	12.79	-0.533	6.5	231
8	5.04	3.61	3.84	17.30	4.89	9.63	14.68	-0.544	6.53	478
9	4.84	3.16	3.4	17.08	4.93	9.12	14	-0.542	6.5	452
10	5.27	3.18	3.39	18.36	4.79	9.06	14.29	-0.552	6.6	325
11	3.91	3.48	3.73	16.73	4.76	9.37	13.44	-0.538	6.52	297
12	3.83	3.38	3.63	10.65	4.91	9.33	13.21	-0.551	6.56	499
13	4.75	3.33	3.57	10.46	5.07	9.46	14.25	-0.542	6.45	299

14	6.00	2.6	2.84	14.81	4.69	8.36	14.36	-0.541	6.5	417
15	3.65	2.9	3.12	10.82	4.68	8.54	12.28	-0.522	6.48	260

Table S3. The main biochemical parameters and component composition of milk samples from 13 black-and-white cows (group 3).

№	TFP, %	TP1, %	TN2, %	TAWSA mg/g	Lactose, %	SNF, %	TDM, %	FP, ºC	pH, units	SCC, 10^3 cell/ml
1	4.68	4.06	4.27	17.70	3.51	8.58	13.3	-0.526	6.5	687
2	5.61	3.39	3.61	15.30	4.73	9.24	14.88	-0.551	6.49	940
3	5.08	2.87	3.06	15.40	4.55	8.41	13.53	-0.542	6.54	903
4	6.35	3.49	3.72	13.60	4.01	8.61	15.09	-0.546	6.47	973
5	6.46	3.55	3.78	16.70	4.15	8.78	15.28	-0.547	6.42	556
6	5.37	4.57	4.81	14.80	4.55	10.36	15.82	-0.552	6.65	933
7	4.06	3	3.24	16.09	5.14	9.18	13.27	-0.538	6.58	658
8	4.74	3.4	3.63	17.37	4.72	9.2	14.03	-0.540	6.56	910
9	6.08	4.47	4.75	16.46	4.85	10.6	16.76	-0.551	6.68	681
10	4.76	2.48	2.71	22.25	4.43	7.92	12.72	-0.519	6.52	542
11	5.35	3.62	3.85	14.24	4.46	9.3	14.72	-0.544	6.42	809
12	4.86	3.42	3.62	13.89	4.97	9.46	14.35	-0.544	6.67	973
13	6.84	4.26	4.54	14.79	4.57	10.07	17.02	-0.541	6.49	903

Table S4. The main biochemical parameters and component composition of milk samples from 12 black-and-white cows (group 4).

№	TFP, %	TP1, %	TN2, %	TAWSA mg/g	Lactose, %	SNF, %	TDM, %	FP, ºC	pH, units	SCC, 10^3 cell/ml
1	5.00	4.16	4.39	13.00	4.05	9.35	14.47	-0.519	6.58	3149
2	4.92	3.47	3.7	5.80	4.92	9.48	14.54	-0.542	6.56	-*
3	5.13	2.38	3.6	12.50	4.47	8.93	14.18	-0.545	6.52	-*
4	6.29	3.97	4.25	17.80	3.63	8.68	15.06	-0.524	6.54	1567
5	6.97	3.35	3.55	11.30	4.63	9	14.01	-0.535	6.62	1326
6	5.34	4.24	4.48	14.20	4.19	8.47	13.88	-0.517	6.44	7493

7	4.71	3.49	3.72	20.30	4.3	8.83	13.61	-0.537	6.42	4803
8	4.69	3.18	3.45	16.90	4.15	8.44	16.27	-0.517	6.36	2614
9	6.28	2.94	3.18	18.73	4.69	8.72	14.08	-0.541	6.47	1358*
10	4.86	3	3.21	13.08	4.87	8.99	13.89	-0.538	6.65	1004
11	3.80	3.57	3.83	18.68	5.04	9.79	13.69	-0.554	6.57	2466
12	4.21	2.79	3.01	12.70	4.71	8.44	12.71	-0.536	6.52	4910

Notes: total fat percentage (TFP); true protein percentage (TP1); total nitrogen percentage (TN2); lactose; solids-not-fat (SNF); total dry matter (TDM); freezing point (FP); somatic cell count (SCC).
